# Inhibition of Brd4 by JQ1 Promotes Functional Recovery From Spinal Cord Injury by Activating Autophagy

**DOI:** 10.3389/fncel.2020.555591

**Published:** 2020-09-02

**Authors:** Yao Li, Jie Xiang, Jing Zhang, Jiahao Lin, Yaosen Wu, Xiangyang Wang

**Affiliations:** ^1^Department of Orthopaedics, The Second Affiliated Hospital and Yuying Children’s Hospital of Wenzhou Medical University, Wenzhou, China; ^2^Department of Orthopaedics, Taizhou Hospital of Zhejiang Province, Taizhou, China; ^3^Molecular Pharmacology Research Center, School of Pharmaceutical Science, Wenzhou Medical University, Wenzhou, China

**Keywords:** BRD4, JQ1, autophagy, functional recovery, spinal cord injury

## Abstract

Spinal cord injury (SCI) is a destructive neurological disorder that is characterized by impaired sensory and motor function. Inhibition of bromodomain protein 4 (Brd4) has been shown to promote the maintenance of cell homeostasis by activating autophagy. However, the role of Brd4 inhibition in SCI and the underlying mechanisms are poorly understood. Thus, the goal of the present study was to evaluate the effects of sustained Brd4 inhibition using the bromodomain and extraterminal domain (BET) inhibitor JQ1 on the regulation of apoptosis, oxidative stress and autophagy in a mouse model of SCI. First, we observed that Brd4 expression at the lesion sites of mouse spinal cords increased after SCI. Treatment with JQ1 significantly decreased the expression of Brd4 and improved functional recovery for up to 28 day after SCI. In addition, JQ1-mediated inhibition of Brd4 reduced oxidative stress and inhibited the expression of apoptotic proteins to promote neural survival. Our results also revealed that JQ1 treatment activated autophagy and restored autophagic flux, while the positive effects of JQ1 were abrogated by autophagy inhibitor 3-MA intervention, indicating that autophagy plays a crucial role in therapeutic effects Brd4 induced by inhibition of the functional recovery SCI. In the mechanistic analysis, we observed that modulation of the AMPK-mTOR-ULK1 pathway is involved in the activation of autophagy mediated by Brd4 inhibition. Taken together, the results of our investigation provides compelling evidence that Brd4 inhibition by JQ1 promotes functional recovery after SCI and that Brd4 may serve as a potential target for SCI treatment.

## Introduction

Spinal cord injury (SCI) is one of the most destructive neurological traumas and typically results from vehicle accidents, falls and violence. SCI is characterized by impaired sensory and motor function, which in some instances can be life-threatening, resulting in great challenges associated with health-related quality of life and lifetime healthcare costs (Badhiwala et al., 2019; Courtine and Sofroniew, 2019). The physiological progression of SCI involves two phases, the primary injury, which is caused by an immediate mechanical insult, then the secondary injury, which arises following the initial damage that triggers a series of molecular events, including the inflammatory response, endoplasmic reticulum stress, mitochondrial dysfunction, oxidative stress and axonal demyelination, exacerbating the initial injury and affecting neural repair for a several weeks (Silva et al., 2014; Brommer et al., 2016). Currently, separate or combined methods to target secondary responses may be beneficial for maintaining neural tissue survival and improving long-term functional recovery (Wang et al., 2018; Li et al., 2019; Zheng et al., 2019).

As a member of the bromodomain and extraterminal domain (BET) family, bromodomain protein 4 (Brd4) harbors an extraterminal domain and two N-terminal bromodomains and plays a crucial role in transcriptional regulation, initiation and elongation (Donati et al., 2018). Brd4 was first discovered as a cell cycle progression regulator of chromatin stability, which is essential for embryogenesis and cell identity determination (Yang et al., 2008; Liu et al., 2014). Intriguingly, Brd4 has emerged as a promising target for the treatment of various diseases. For instance, Brd4 has been reported to modulate pathological cardiac remodeling, and its inhibitor has potential for the treatment of heart failure (Spiltoir et al., 2013). Brd4 has also been shown to be involved in memory function and age-related memory impairment, making it a suitable target to treat neurodegenerative disease (Magistri et al., 2016; Benito et al., 2017). Furthermore, Brd4 inhibition has been shown to reduce blood–brain barrier damage and stroke volume, improving sensory and motor function after stroke (Demars et al., 2019). Interestingly, the results of several studies have also suggested that Brd4 inhibition is beneficial for functional recovery after SCI, and the underlying mechanism was shown to involve the inhibition of neural inflammation (Rudman et al., 2018; Sánchez-Ventura et al., 2019; Wang et al., 2019a). However, the effect of Brd4 inhibition in the spinal cord has been not well-elucidated, with current data not allowing for definitive conclusions to be made.

Autophagy is a conserved catabolic and lysosomal-dependent process for macromolecular circulation that disposes of damaged organelles and aggregated or misfolded proteins (Parzych and Klionsky, 2014). The results of numerous studies have suggested that basal autophagy is essential for maintaining neuronal homeostasis but that autophagic flux is inhibited in neurons after SCI (Sarkar et al., 2014; Galluzzi et al., 2016). Previous studies have proved that restoring autophagic flux can reduce oxidative and endoplasmic reticulum stress, which is beneficial for neuron survival and functional recovery after SCI (Liu et al., 2015; Su et al., 2016). In addition, Brd4 has been proposed to be a repressor of autophagic activity, and the protective effects of Brd4 inhibition on cells under nutrient deprivation is due to the activation of autophagy (Sakamaki et al., 2017; Wen and Klionsky, 2017). However, autophagy also can induce cell death, and the biological role of Brd4 in neural autophagy and whether Brd4 inhibition is beneficial for neuron survival after SCI requires further investigation.

Given that Brd4 is involved in crucial physiological functions and the development of various pathologies, several efficient small molecule inhibitors for blocking BET by binding to acetylated residues have been identified. One of these BET inhibitors, JQ1, is associated with high specificity for Brd4 inhibition and has been shown to exert protective effect in several diseases, including arthritis, heart remodeling, Alzheimer’s disease and stroke (Spiltoir et al., 2013; Benito et al., 2017; An et al., 2018; Demars et al., 2019).

In the present study, we evaluated the effect of Brd4 inhibition by JQ1 on neural oxidative stress, apoptosis and autophagy after SCI. In addition, the mechanism by which JQ1 improves the survival of neurons was also elucidated. Overall, using conventional behavioral and molecular methods, we showed that JQ1 administration efficiently inhibits Brd4 expression in neurons and promotes functional recovery from SCI through activation of the AMPK-mTOR signaling pathway, which results in the restoration of autophagic flux and subsequently attenuates neural oxidative stress and apoptosis after SCI. Our observations provide important evidence that JQ1 may have potential for use in SCI management.

## Materials and Methods

### Animals and Ethics Statement

**FIGURE 1:**
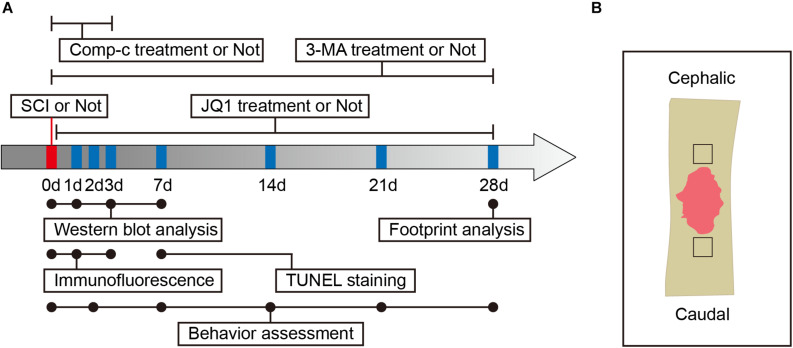
Experimental protocols. **(A)** Timeline diagram of SCI, drugs treatment and experimental analysis in mice. **(B)** A schematic of a longitudinal spinal cord section after SCI, the area surrounded by the black dotted line is the region of interest for images capture.

Adult male C57BL/6 mice (7–8 week-old, 20–25 g) were obtained from Wenzhou Medical University and housed in OptiMICE Rotary Experimental Animal Cage System (C89100, Animal Care Systems, United States) under standard conditions (temperature: 23 ± 2°C, humidity: 50 ± 5%, 12 h light/dark cycle). All mice had free access to water and food. All animal experiments are shown in a flowchart in Figure 1A and were approved by the Animal Care and Use Committee of Wenzhou Medical University (wydw2018-0043) and strictly complied with the National Institutes of Health Guide for the Care and Use of Laboratory animals.

### Reagents and Antibodies

Regarding reagents used in the present study, (+)-JQ1 was purchased from Meilunbio (MB7243, Shandong, China); bafilomycin A1 (Baf-A1, HY100558) and dorsomorphin (Comp-c, HY13418A) were purchased from MCE (Princeton, NJ, United States); tert-Butyl hydroperoxide solution (TBHP,416665), 3-methyladenine (3-MA, M9281) was purchased from Sigma-Aldrich (St. Louis, MO, United States); and 4,6-diami- dino-2-phenylindole (DAPI) was obtained from Beyotime (P0131, Shanghai, China). Primary antibodies and the companies from which they were obtained were as follows: antibodies against BRD4 (ab128874), NeuN (ab104224), beta III tubulin (Tuj1, ab18207), Bax (ab199677), beclin-1 (ab210498), Lamp2 (ab13524), p62 (ab56416), p-ULK1 (ab133747), p-AMPK (ab23875) were obtained from Abcam (Cambridge, MA, United States); antibodies against Ace-tubulin (5335), cytochrome c (Cyt-c, 11940), ATG5 (12994), ATG12 (4180), AMPK (2532), p-AMPK (2537), mTOR(2983), p-mTOR(5536) were obtained from Cell Signaling Technology (Danvers, MA, United States); an antibody against Tyr-tubulin (T9028) was obtained from Sigma-Aldrich; antibodies against SOD1 (10269-1), HO-1 (10701-1), Bcl-2 (26593-1), GAPDH (10494-1), and ULK1 (20986-1) were obtained from Proteintech Group (Rosemont, IL, United States); an antibody against cleavedcaspase-3 (AF7022) was obtained from Affinity Biosciences (Cincinnati, OH, United States); and an antibody against LC3B (NB600-1384) was obtained from NOVUS Biologicals (Littleton, CO, United States). Horseradish peroxidase (HRP)-conjugated IgG secondary antibodies against mouse, rat and rabbit were purchased from Proteintech Group; Alexa Fluor FITC or Cy5 donkey anti-rabbit/mouse/rat secondary antibodies were purchased from Abcam.

### Establishment of a Contusive SCI Model

Mice were intraperitoneally injected with 4% (w/v) pentobarbital sodium (40 mg/kg) for anesthesia. Moderate contusive SCIs were established at the T9/10 level by a weight drop. In brief, after shaving hair and disinfection, a laminectomy was performed after incision of the skin and muscle adjacent to the spinous processes, the exposed spinal cord was subjected to a moderate crush injury using a 10 g weight steel rod from a height of 20 mm according to a previous study (Zheng et al., 2019). Then, the muscle and skin was sutured with 5-0 silk, after which mice received 0.5 mL of normal saline and were placed on a warm blanket (37°C) for post-operative recovery. For the control group, a laminectomy was performed without crush injury. Bladders were manually expressed twice daily until the mice regained normal bladder function.

### Drug Administration and Mouse Groups

JQ1 was initially dissolved at 50 mg/mL in dimethyl sulfoxide (DMSO, ST038, Beyotime) and stored at −20°C before being diluted with vehicle solution for injection. Mice received 50 mg/kg/day of JQ1 solution by intraperitoneal injection for Brd4 inhibition (Magistri et al., 2016). To inhibit autophagy of the spinal cord, mice were administered 15 mg/kg/day of 3-MA solution by intraperitoneal injection (Wang et al., 2016). To inhibit AMPK, mice were administered 10 mg/kg/day of Compound c solution by intraperitoneal injection (Yu et al., 2008). For the control group, mice were administered an equal volume of solution following the same protocol described above. According to the experimental settings, a 150 mice were randomly divided into six groups: a Control group (*n* = 30), a JQ1 group (*n* = 10), an SCI group (*n* = 45), an SCI + JQ1 group (*n* = 30), an SCI + JQ1 + 3-MA group (*n* = 25), and an SCI + JQ1 + Comp-c group (*n* = 10). The time points of drug administration are presented in the experimental flowchart in Figure 1A.

### Cells and Primary Cortical Neuron Culture

PC12 cells (Cell Storage Center of Wuhan University, Wuhan, China) were cultured and propagated in RPMI1640 medium (21870076, Gibco, Carlsbad, CA, United States) supplemented with 10% fetal bovine serum (p30-3301, PAN-Biotech GmbH, Baghlia, Germany) and 1% penicillin/streptomycin solution (15140122, Gibco) at 37°C under an atmosphere with 5% CO_2_. PC12 cells were cultured for 3 days in the presence of 50 ng/mL β-NGF (11050-HNAC, SinoBiological, Beijing, China) to induce their differentiation into neuron-like cells. The culture medium was replaced with fresh medium every 3 days.

Primary cortical neurons were obtained from postnatal Day 0 (P0) mouse pups by dissecting the cerebral cortex as described previously (Li et al., 2019). Primary cortical neurons were seeded in poly-D-lysine-coated 12-well plates at a density of 1–2 × 10^5^ cells per well and cultured in Neurobasal medium (21103049, Gibco) supplemented with 2% B27 (17504044, Gibco) and 1% glutamine (25030081, Gibco) at 37°C under an atmosphere with 5% CO_2_. The culture medium was replaced with fresh medium on the second day and every 3 days.

### Cell Viability Assay

Cell viability was assessed using the Cell Counting Kit-8 (CCK-8) assay (CK04, Dojindo Laboratories, Kyushu, Japan) according to the manufacturer’s protocol. PC12 cells were digested with 0.25% Trypsin-EDTA (12605028, Gibco), after which the cell suspension was seeded in a 96-well plate (1 × 10^4^ cells per well) and incubated at 37°C under an atmosphere with 5% CO_2_ with complete medium for 24 h. Then, the cells were treated with drugs according to the experimental design. After reagent treatment, 10 μL of CCK-8 solution was added on to each well, and the cells were cultured for 2 h. Subsequently, the absorbance of each well was measured at 450 nm with a microplate reader.

### Locomotion Recovery Assessment

Locomotion recovery assessments, including the Basso mouse scale (BMS), balance beam test, inclined plane test and footprint analysis, were performed at baseline and 2, 7, 14, 21, and 28 days after injury. BMS was used to evaluate the hind limb motor function and the coordination in movement by observing the mobility of the hind limb-ankle joint, coordination, paw posture, trunk stability, and tail posture (Basso et al., 2006). The mice were placed in an open field and allowed to move freely for 5 min and observed for a 2 min for scoring. The balance beam test was evaluated by recording the walking time of mice on the modified beam (Doeppner et al., 2014). The beam was suspended 15 cm high and 110 cm long with gradually reduced width (12-mm width at the beginning and 5-mm width at the end), where the result of test is the time that passed from the beginning to the end. Mice that failed to reach the destination or the walking time exceed 60 s were both recorded as 60 s. The inclined plane test evaluated the strength of hind limbs by recording the maximum board angle (Li et al., 2019). In this test, the mice were placed on a board with a rubber surface, after which the angle at which the mouse could not maintain its position for 5 s without falling was defined the maximum angle and recorded. The footprint analysis was performed by first dipping the hind limbs of the animal in red dye and its fore limbs in blue dye, after which the mice were allowed to walk across a narrow box to record the motion trajectories (Zheng et al., 2019).

### Tissue Preparation

Mice were euthanized by an overdose of 8% (w/v) pentobarbital sodium (40 mg/kg) at the indicated time points followed by ventricular perfusion with normal saline. Then, 10-mm long sections of spinal cord centered around the epicenter of the lesion sites were harvested and stored at −80°C immediately for later preparation for western blot analysis. For staining, mice were prepared by ventricular perfusion with normal saline followed by 4% paraformaldehyde (PFA), after which a 10-mm long of spinal cord was dissected out and fixed in 4% PFA for 24 h. The post-fixed spinal cord was then dehydrated, embedded in paraffin, and mounted onto 0.5-μm slides for subsequent staining.

### Western Blot Analysis

Extracted proteins from the animals or cell lysates were quantified using the Bradford assay (ab119216, Abcam), and equivalent amounts of protein (80 μg *in vivo*, 30 μg *in vitro*) were separated by sodium dodecyl sulfate polyacrylamide gel electrophoresis (SDS-PAGE) and then transferred onto a polyvinylidene fluoride membranes (1620256, Bio-Rad, Berkeley, CA, United States). The PVDF membranes were blocked with 5% skimmed milk in Tris-buffered saline with 0.1% Tween-20 (TBST) for 90 min at room temperature and then incubated overnight at 4°C with primary antibodies against the following proteins: Brd4 (1:1000), SOD1 (1:500), Cyt-c (1:1000), HO-1 (1:500), Bax (1:1000), Bcl-2 (1:300), cleavedcaspase-3 (1:1000), LC3 (1:1000), Atg5 (1:1000), Atg12 (1:1000), beclin-1 (1:1000), p62 (1:1000), p-AMPK (1:500), AMPK (1:1000), p-mTOR (1:500), mTOR (1:1000), P-ULK1 (1:500), ULK1 (1:300), and GAPDH (1:1000). The following day, the membranes were washed with TBST and incubated with secondary antibodies (1:1000) for 90 min at room temperature. Then, the membranes were visualized using an ECL Plus Reagent kit (BL520A, Biosharp, Anhui, China) and a ChemiDocXRS + Imaging System (Bio-Rad). The bands were quantified using densitometric measurement with Quantity-One software.

### Immunofluorescence Staining

The deparaffinized, rehydrated sections of spinal cord and neurons fixed in 4% PFA were blocked with 5% bovine serum albumin (A7030, Sigma, Shanghai, China) for 30 min at room temperature and then incubated overnight at 4°C with primary antibodies against the following proteins: Brd4 (1:1000), Tuj1 (1:1000), Ace-tubulin (1:1000), Tyr-tubulin (1:1000), cleaved caspase-3 (1:1000), LC3 (1:1000), Lamp2 (1:1000), p-AMPK (1:500) and NeuN (1:1000). On the 2nd day, sections and fixed cells were incubated with Alexa Fluor FITC-, TRITC- or Cy5-conjugated donkey anti-rabbit/mouse/rat secondary antibodies (1:1000) for 60 min at 37°C, after which cellular nuclei were labeled with DAPI (36308ES20, Yeasen Biochemical, Shanghai, China). Images of different mice from the region of interest around the lesion margin (Figure 1B) and cells were blindly captured using a Nikon ECLIPSETi microscope (Nikon, Tokyo, Japan) with the same imaging setup.

### TUNEL Staining

A terminal deoxynucleotidyl transferase dUTP nick-end labeling (TUNEL) assay kit was used following the manufacturer’s instructions. Briefly, the deparaffinized, rehydrated sections of spinal cord were incubated in 0.1% Triton X-100 for 30 min and then stained with 50 μL of reagent from an *In Situ* Cell Death Detection Kit (40307ES60, Yeasen Biochemical), after which the sections were stained with DAPI and imaged with a Nikon ECLIPSE Ti microscope.

### Statistical Analysis

All the data are presented as the mean ± standard deviation from at least three independent experiments. Statistical analyses were performed using Student’s *t*-test or the Mann–Whitney rank sum test for comparisons between two groups, while two-way analysis of variance (two-way ANOVA) followed by a Tukey’s test or Kruskal–Wallis ANOVA based on ranks followed by Dunn’s *post hoc* test was used for pair-wise comparisons of multiple groups. Comparisons at multiple time points for the behavior test were analyzed with a repeated measure two-way ANOVA followed by an LSD test for between-group comparisons. A *p*-value < 0.05 was considered to be significant.

## Results

### Brd4 Expression Is Increased in Neurons After Injury

**FIGURE 2:**
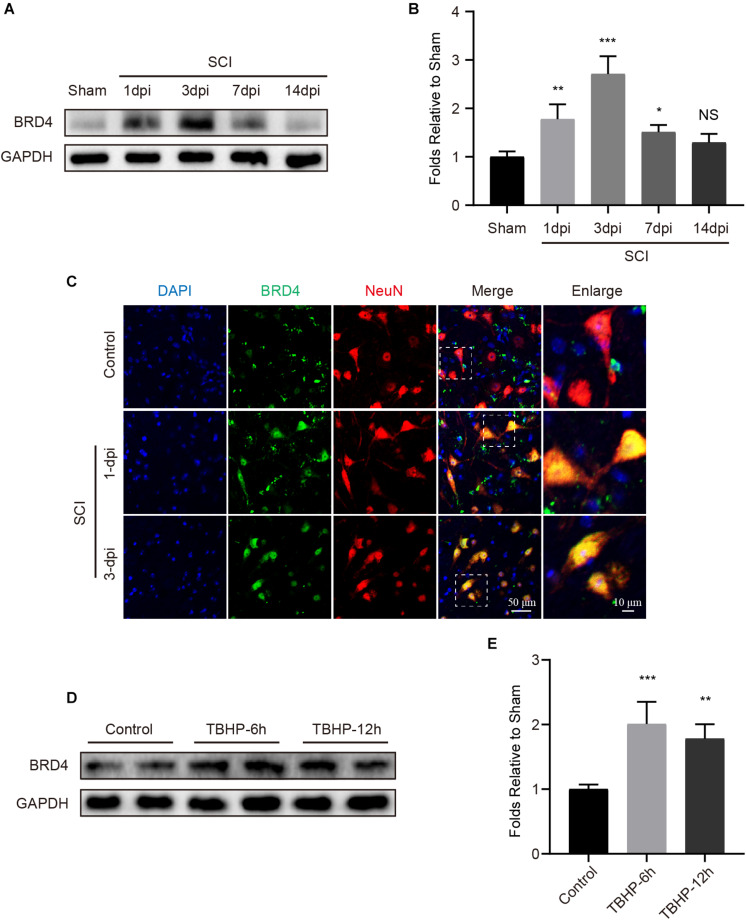
Brd4 expression is increased after injury insult both *in vivo* and *in vitro*. **(A,B)** Western blotting and quantification of the changes of Brd4 expression in mice at several time points after SCI, *n* = 5. **(C)** Representative immunofluorescence of Brd4 (green) puncta in neuron (NeuN, red) in mice at different time points after SCI, *n* = 5. Scale bar = 50 μm, scale bar (enlarged) = 10 μm. **(D,E)** Western blotting and quantification of the changes of Brd4 expression in primary cortical neurons at 6 and 12 h after TBHP (50 μM) treatment, *n* = 4. GAPDH was the loading control. *P < 0.05, **P < 0.01, ***P < 0.001 and NS (not significant) VS. control group or sham group. Data were expressed as means ± SD.

To assess Brd4 expression in the spinal cord after injury, we evaluated the Brd4 protein levels in a time-dependent manner. Western blot results indicated that the levels of Brd4 expression were remarkably increased and peaked at the 3rd day after injury (Figures 2A,B). Additionally, immunofluorescence staining results of the lesion margin (Figure 1B) revealed that Brd4 was typically located in neurons (Figure 2C). To further confirm that Brd4 is present in neurons, the cortical neurons were separately cultured and treated with TBHP, which is a well-established ROS donor used to simulate neural injury *in vitro* (Wu et al., 2016). The results showed that neurons subjected TBHP stimulation contained higher levels of Brd4 compared to that observed in the control group (Figures 2D,E). Thus, these results suggest that Brd4 expression is increased in neurons after SCI and may be involved in neural modulation.

### Inhibition of Brd4 by JQ1 Enhances Functional Recovery After SCI

**FIGURE 3:**
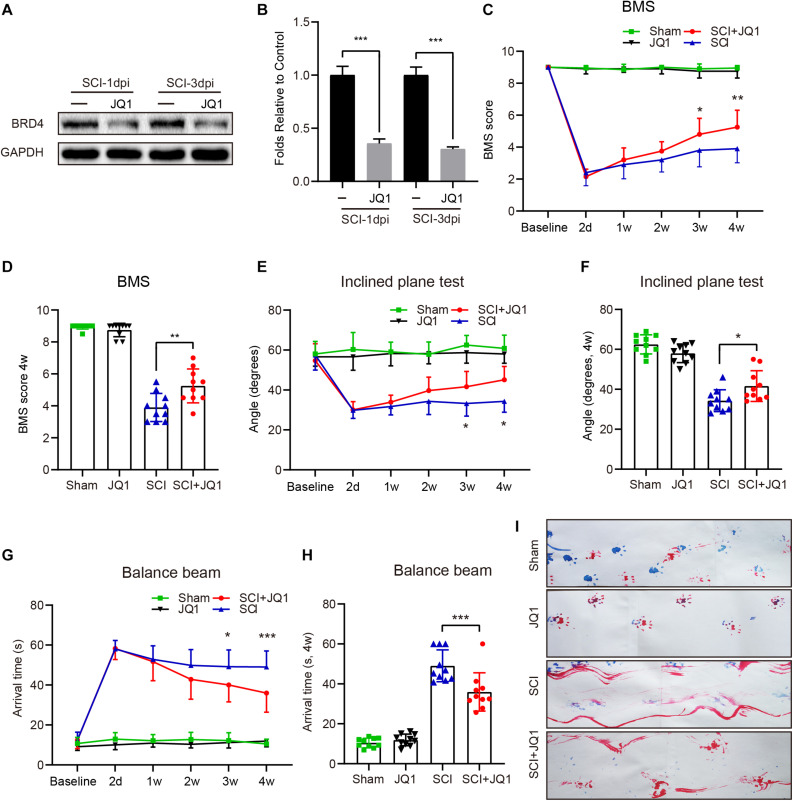
Pharmacological inhibition of Brd4 by JQ1 promotes functional recovery after SCI. **(A,B)** Western blotting and quantification of the Brd4 expression in mice treated with JQ1 at 1 and 3 days after SCI, *n* = 5. **(C,D)** Statistical analysis of BMS in sham and injured mice treated with or without JQ1 at different time points, *n* = 10. **(E,F)** Statistical analysis of angle of inclined plane test in each group at different days after SCI, *n* = 10. **(G,H)** Statistical analysis of arrival time of balance beam test in each group at different days after SCI, *n* = 10. **(I)** Representative footprint analysis pattern in the different groups at 28 days after SCI (blue, fore limbs; red, hind limbs), *n* = 10. GAPDH was the loading control. *P < 0.05, **P < 0.01, ***P < 0.001. Data were expressed as means ± SD.

Previous studies have shown that inhibiting Brd4 represents a potential therapeutic approach for several diseases (Benito et al., 2017; An et al., 2018). Thus, JQ1 was used to selectively inhibit Brd4 expression. As expected, mice treated with JQ1 showed decreased levels of Brd4 at both 1 and 3 days after injury (Figures 3A,B). Moreover, a 4-week period of behavioral tests, which included the BMS, balance beam and inclined plane tests were performed to determine the potential effect of Brd4 inhibition by JQ1 after SCI. The BMS test results revealed that all mice showed significantly declined scores at 2 days after SCI and gradually recovered several weeks thereafter. Notably, mice suffering from SCI presented higher scores following JQ1 treatment compared to injured mice with no intervention at 3 and 4 weeks after SCI (Figures 3C,D). The results of the inclined plane test also revealed that SCI mice treated with JQ1 exhibited significantly greater hind limb strength than those in the SCI group at 3 and 4 weeks after SCI (Figures 3E,F). Similar to the above tests, the balance beam test results also indicated that injured mice treated with JQ1 performed better in motor coordination and balance tests than those in the injured group at the later stages of injury (Figures 3G,H). In addition, the representative footprint analysis results showed that mice treated with JQ1 exhibited greater restoration of hind leg movement with coordinated crawling after SCI (Figure 3I). Collectively, these results demonstrated that inhibiting Brd4 by JQ1 treatment has a promoting effect on SCI recovery.

### JQ1 Treatment Maintains Neuronal Homeostasis After Injury *in vitro*

**FIGURE 4:**
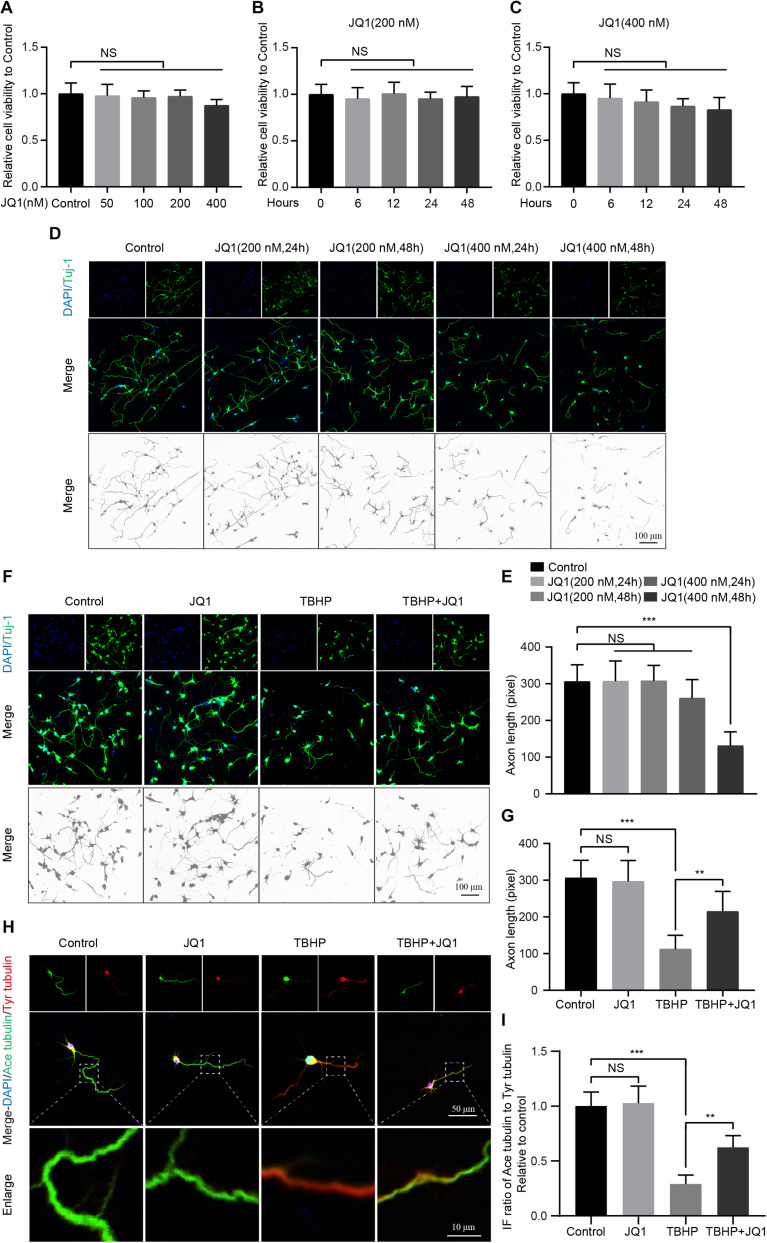
JQ1 treatment improves primary cortical neuron homeostasis after TBHP insult. **(A)** The cell viability of PC12 cells at 24 h after different concentrations of JQ1 treatment, *n* = 6. **(B,C)** The cell viability of PC12 cells treated with JQ1 at concentration with 200 and 400 nM in a time dependent manner, *n* = 6. **(D,E)** Representative neuron morphology stained with Tuj1 (green) and quantitative analysis of axonal length of neuron under different concentrations of JQ1 at different time points, *n* = 24. Scale bar = 50 μm. **(F,G)** Representative neuron morphology stained with Tuj1 (green) and quantitative analysis of axonal length of neuron in each group, neuron of above groups were treated with JQ1 (200 nM), TBHP (50 μM) or co-treated with JQ1 (200 nM) and TBHP (50 μM) for 6 h, respectively, *n* = 24. Scale bar = 50 μm. **(H,I)** Representative images of axonal microtubule stabilization by stained with ace-tubulin (green) and tyr-tubulin (red) and quantitative analysis of ratio of ace-tubulin to tyr-tubulin in each group as mentioned above, *n* = 10. Scale bar = 50 μm, scale bar (enlarged) = 10 μm. *P < 0.05, **P < 0.01, ***P < 0.001, and NS (not significant). Data were expressed as means ± SD.

To further evaluate the effect of JQ1 on neurons, the viability and growth neurons were monitored. First, the cytotoxic effects of different concentrations of JQ1 on PC12 cells were determined, and the results indicated that JQ1 treatment had no significant cytotoxicity at concentrations up to 400 nM after 24 h (Figure 4A). The cytotoxic effects of JQ1 at concentrations of 200 and 400 nM were evaluated in a time-dependent manner, and the results also showed no obvious negative effects from over the course of the assay for both concentrations (Figures 4B,C). Furthermore, we analyzed the morphology of primary cortical neurons by staining Tuj1, and the treatments with different concentrations of JQ1 caused unnoticeable alterations in neural morphology and axon length except for the 48 h treatment with 400 nM JQ1 (Figures 4D,E). In addition, pretreatment with JQ1 significantly reversed TBHP induced cytotoxicity and axon damage at a concentration of 200 nM (Figures 4F,G). Importantly, microtubule stabilization is one of the references of axon growth and neural homeostasis (Li et al., 2018, 2019), and JQ1 treatment remarkably reversed the TBHP-mediated increase in the ratio of ace-tubulin to tyr-tubulin and axon retraction (Figures 4H,I). Thus, appropriate JQ1 treatment benefits neuron growth and homeostasis in response to injury insult.

### JQ1 Attenuates Oxidative Stress and Apoptosis After SCI

**FIGURE 5:**
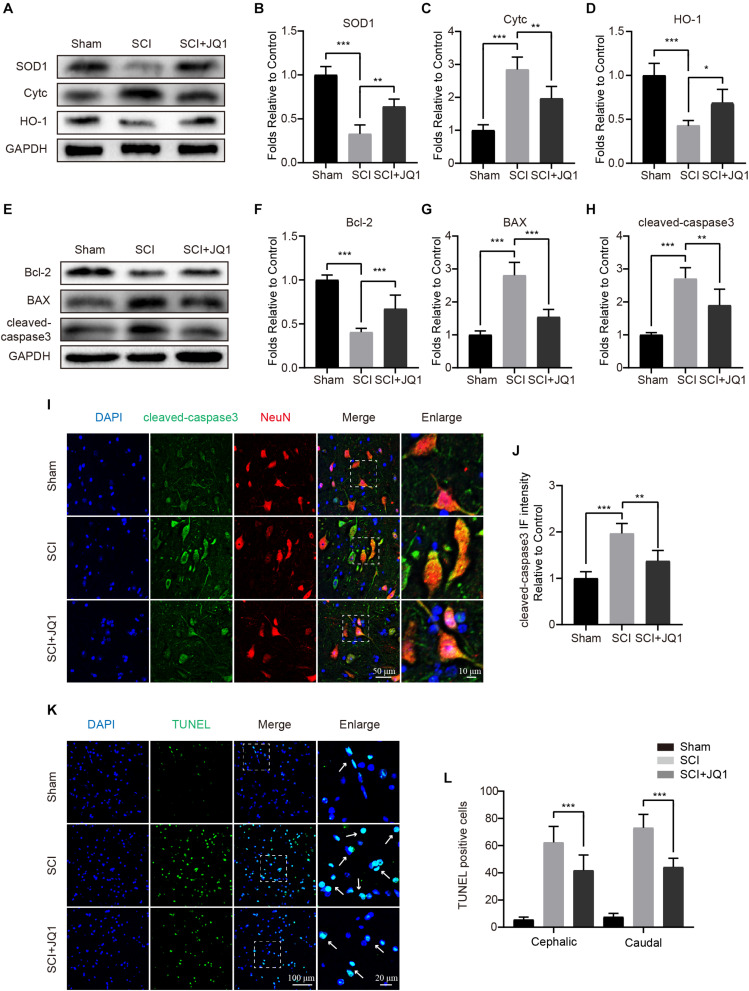
JQ1 reduces oxidative stress and apoptosis after SCI. **(A–D)** Western blotting and quantification of SOD1, Cytc and HO-1 expression in each group at 3 days after SCI in mice, *n* = 5. **(E–H)** Western blotting and quantification of Bcl-2, BAX and cleaved caspase-3 expression in each group at 3 days after SCI in mice, *n* = 5. **(I)** Representative immunofluorescence co-stained with cleaved caspase-3 (green) and NeuN (red) at 3 days after SCI in mice. Scale bar = 50 μm, scale bar (enlarged) = 10 μm. **(J)** The quantitative assessment of the fluorescence intensity of cleaved caspase-3 in each group, *n* = 5. **(K)** Representative immunofluorescence staining for TUNEL (green) from the spinal cord section in each group at 7 days after injury. Scale bar = 100 μm, scale bar (enlarged) = 20 μm. **(L)** The quantification of TUNEL-positive cells from cephalic and caudal area around lesion site, *n* = 10. GAPDH was the loading control. *P < 0.05, **P < 0.01, ***P < 0.001. Data were expressed as means ± SD.

As JQ1 was shown to exert positive effects in resisting TBHP stimulation, we subsequently evaluated whether JQ1 is sufficient to reduce cell death and oxidative stress after SCI. The western blot results showed that JQ1 treatment effectively enhanced the levels of SOD1 and HO-1, both of which are crucial enzymes involved in protecting against oxidative stress, and JQ1 treatment notably decreased the expression of Cytc (Figures 5A–D). Consistent with the results obtained for spinal cords, JQ1 also enhanced the levels of SOD1 and HO-1 in PC12 cells subjected to TBHP (Supplementary Figures 1A–C). To evaluate apoptosis levels, western blot, immunofluorescence, and TUNEL staining analyses were performed to evaluate the levels of apoptotic proteins. As shown in Figures 5E–H, decreased levels of Bax and cleaved caspase-3, and increased level of Bcl-2 were observed after JQ1 treatment for SCI mice. Cleaved caspase-3 was observed to be localized to neurons in immunofluorescence assays, illustrating that the JQ1-treated mice in the SCI group presented a decreased intensity of cleaved caspase-3 compared with that observed in the SCI group (Figures 5I,J). Similarly, JQ1 treatment resulted in a distinct reduction in the number of TUNEL-positive cells after injury (Figures 5K,L). Taken together, these results suggested that the beneficial effects JQ1 exerts on functional recovery after SCI may be due to the inhibition of oxidative stress and apoptosis.

### JQ1 Enhances Autophagy in Neurons After Injury

**FIGURE 6:**
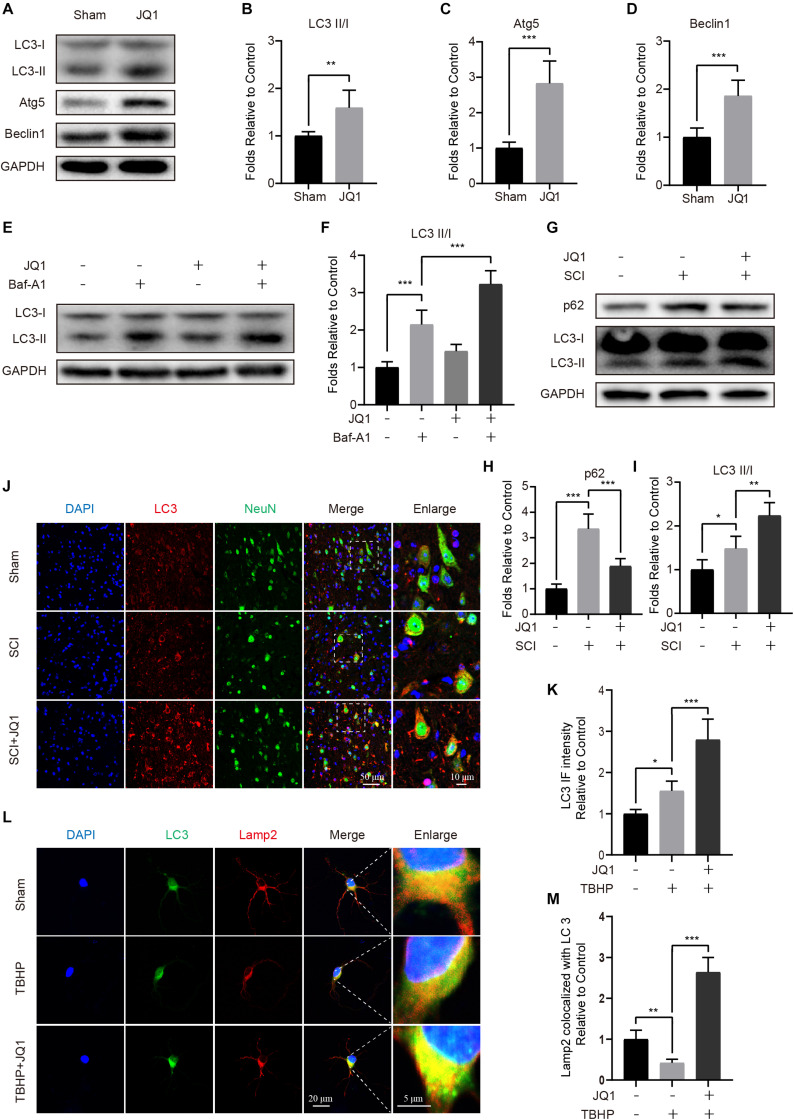
JQ1 enhances autophagy and restores autophagic flux after SCI. **(A–D)** Primary cortical neurons were treated with 200 nM JQ1 for 6 h, the level of LC3, Atg5, and Beclin-1 in each group of neuron were evaluated by western blotting and statistically analyzed, *n* = 5. **(E,F)** Primary cortical neurons were treated with Baf-A1 (100 nM) and JQ1 (200 nM) for 6 h, the level of LC3 in each group of neuron were detected by western blotting and statistically analyzed, *n* = 5. **(G–I)** Western blotting and quantification of p62 and LC3 expression in each group of mice at 3 days after SCI, *n* = 5. **(J,K)** Representative co-staining for LC3 (red) and NeuN (green) in each group of mice at 3 days after SCI, *n* = 5. Scale bar = 50 μm, scale bar (enlarged) = 10 μm. **(L,M)** Primary cortical neurons were treated with TBHP (50 μM) and JQ1 (200 nM) for 6 h, the co-localization of LC3 (green) and Lamp2 (red) were captured by immunofluorescence staining and quantitatively analyzed, *n* = 10. Scale bar = 20 μm, scale bar (enlarged) = 5 μm. GAPDH was the loading control. *P < 0.05, **P < 0.01, ***P < 0.001. Data were presented as means ± SD.

Given the beneficial role of JQ1 in regulating oxidative stress and apoptosis, we reasoned that the potential mechanism involved may be partly associated with the regulation of autophagy. To assess the status of autophagy in neurons, the levels of autophagic-related proteins were detected after JQ1 treatment. Western blot results indicated that JQ1 treatment markedly enhanced the levels of LC3 II/I, Atg5, Atg12-5conjugate and beclin-1 in cortical neurons (Figures 6A–D and Supplementary Figures 2A,B). Autophagy is a dynamic process, and LC3 may decrease due to autolysosome degeneration. Therefore, neurons were treated with Baf-A1 and evaluated to confirm the total amount of LC3. The results showed that Baf-A1 caused an accumulation of LC3-II, and neurons treated with Baf-A1 and JQ1 presented higher level of LC3-II (Figures 6E,F). Furthermore, western blot results revealed increased levels of LC3 II/I and decreased levels of p62 in SCI mice (Figures 6G–I) and PC12 cells (Supplementary Figures 3A–C) treated with JQ1. The immunofluorescence results also showed the increased intensity of LC3 in the JQ-treated SCI group compared with that observed in the SCI group (Figures 6J,K). Consistently, immunofluorescence results showed that the colocalization of LC3 and Lamp2 was decreased in injured neurons, which was improved by JQ1 (Figures 6L,M). Overall, these results suggested that JQ1 enhances the level of autophagy and may be associated with its observed therapeutic effect.

### Inhibition of Autophagy Reverses JQ1-Mediated Protective Effects After SCI

**FIGURE 7:**
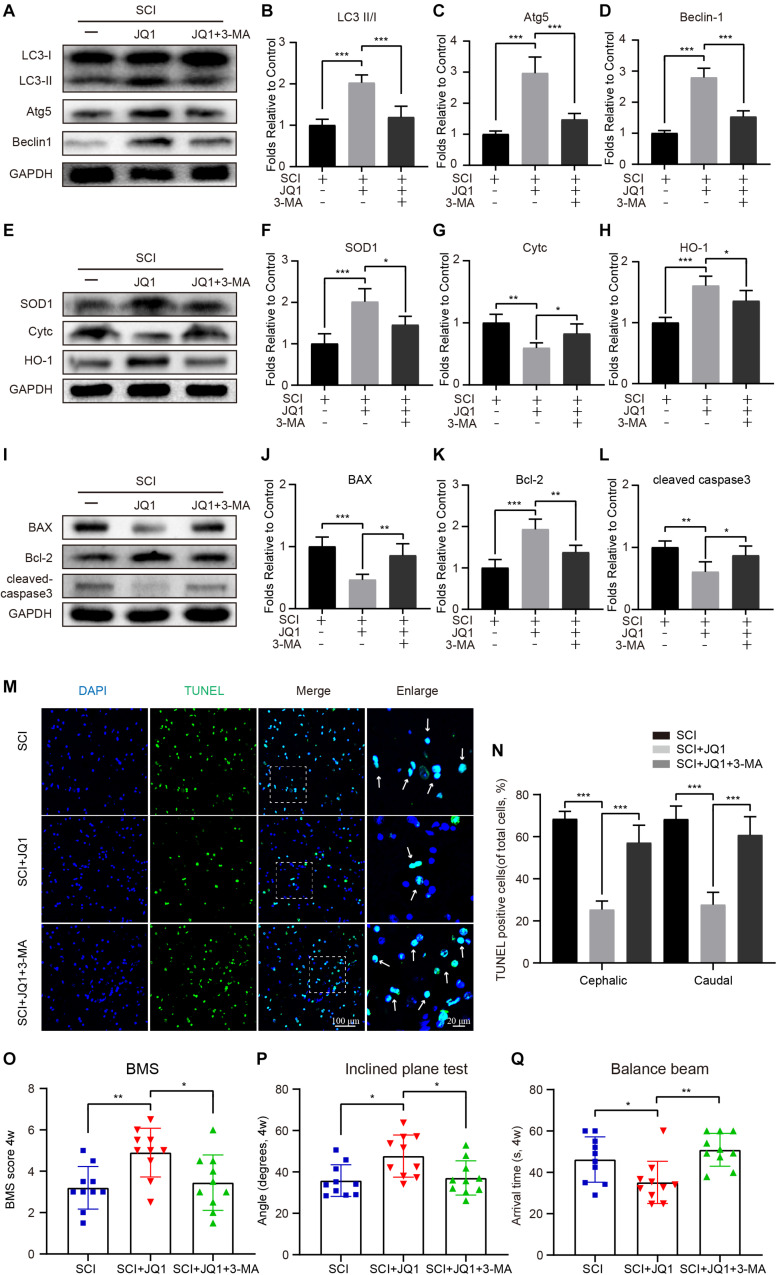
3-MA treatment reverses the effects of JQ1 on oxidative stress, apoptosis and functional recovery. **(A–D)** Western blotting and quantification of LC3, Atg5 and Becliin-1 expression in each group of mice at 3 days after SCI, *n* = 5. **(E–H)** Western blotting and quantification of SOD1, Cytc and HO-1 expression in each group of mice at 3 days after SCI, *n* = 5. **(I–L)** Western blotting and quantification of BAX, Bcl-2 and cleaved caspase-3 in each group of mice at 3 days after SCI, *n* = 5. **(M,N)** Immunofluorescence staining for TUNEL (green) in each group of mice at 7 days after SCI and quantitatively analyzed for the cephalic and caudal area around lesion site, *n* = 10. **(O–Q)** Statistical analysis of BMS, inclined plane test and balance beam test in each group of mice at 28 days after SCI, *n* = 10. Scale bar = 100 μm, scale bar (enlarged) = 20 μm. The mice were treated with or without JQ1 (50 mg/kg) and 3-MA (15 mg/kg) and divided into groups as followed: SCI group, SCI + JQ1 group and SCI + JQ1 + 3-MA group. GAPDH was the loading control.*P < 0.05, **P < 0.01, ***P < 0.001. Data were presented as means ± SD.

To further determine whether the activation of autophagy by JQ1 is beneficial for recovery after SCI, mice were co-treated with 3-MA and JQ1. As expected, 3-MA treatment caused a rapid decrease of autophagic proteins, including LC3 II/I, Atg5, and beclin-1 (Figures 7A–D). In addition, 3-MA treatment observably decreased the expression of SOD1 and HO-1 and increased that of Cytc, as evidenced by western blot results (Figures 7E–H). On the other hand, co-treatment with 3-MA and JQ1 significantly upregulated the expression of cleaved caspase-3 and Bax and downregulated that of Bcl2 compared to that observed after JQ1 treatment (Figures 7I–L). Similarly, the TUNEL staining results also showed that the group of mice treated with 3-MA exhibited more TUNEL-positive cells than that observed in the group of mice treated with JQ1 alone (Figures 7M,N). Furthermore, behavioral tests, including the BMS, inclined plane and balance beam tests revealed that 3-MA pretreatment inhibited the functional recovery of hind limbs in injured mice treated with JQ1 (Figures 7O–Q). Therefore, these data suggest that the JQ1-mediated inhibition of Brd4 is required for the beneficial activation of autophagy.

### AMPK-mTOR Signaling Is Associated With Autophagy Induction Resulting From the JQ1-Mediated Inhibition of Brd4

**FIGURE 8:**
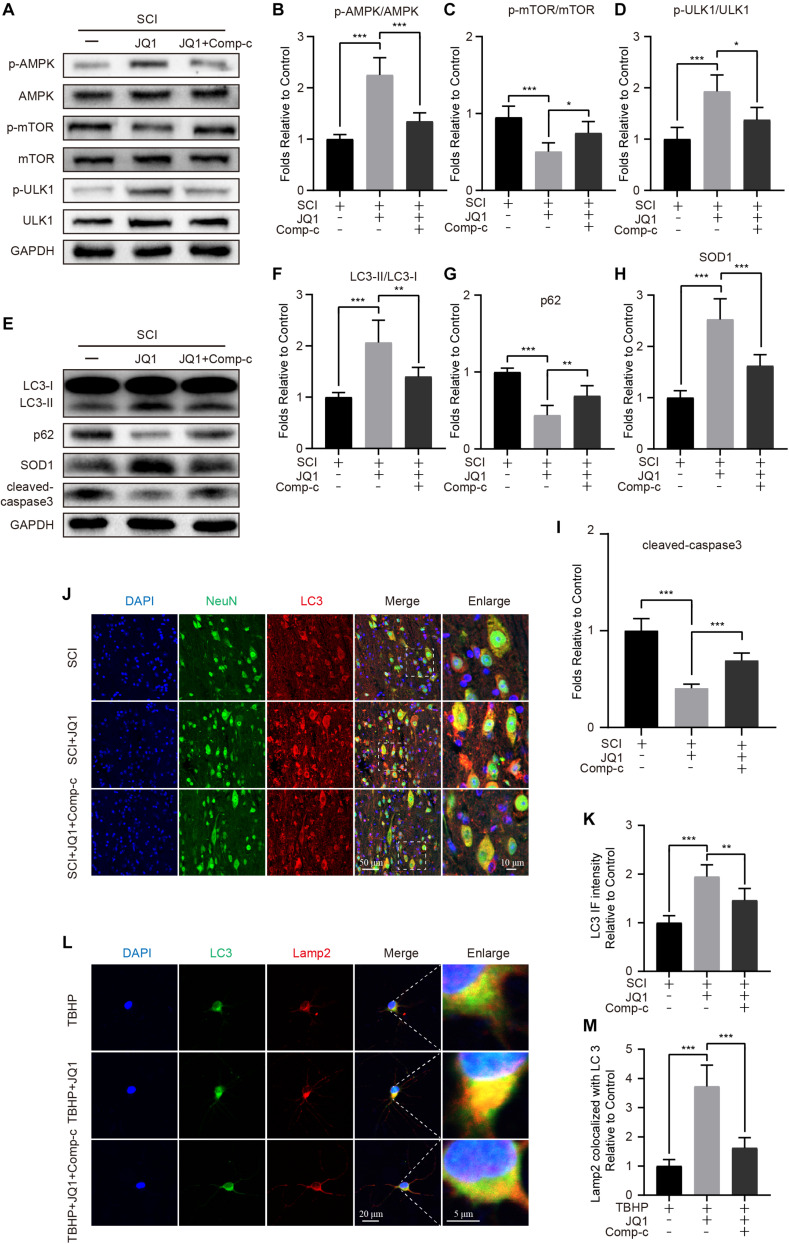
Compound c treatment inhibits JQ1 induced autophagy activation. **(A–D)** Western blotting and quantification of p-AMPK, AMPK, p-mTOR, mTOR, p-ULK1, and ULK1 in each group of mice at 3 days after SCI, *n* = 5. **(E–I)** Western blotting and quantification of LC3, p62, SOD1 and cleaved caspase-3 in each group of mice at 3 days after SCI, *n* = 5. **(J,K)** Immunofluorescence staining for the co-localization of LC3 (red) and NeuN (green) and quantitative analysis in the each group of mice at 3 days after SCI, *n* = 5. Scale bar = 50 μm, scale bar (enlarged) = 10 μm. **(L,M)** Immunofluorescence staining for the co-localization of LC3 (green) and Lamp2 (red) and quantitative analysis in the each group of mice, *n* = 10. Scale bar = 20 μm, scale bar (enlarged) = 5 μm. The mice were treated with or without JQ1 (50 mg/kg) and Compound c (10 mg/kg) and divided into groups as followed: SCI group, SCI + JQ1 group and SCI + JQ1 + Compound c group. The neurons were treated with or without TBHP (50 μM), JQ1 (200 nM) and Compound c (10 μM), and divided into TBHP group, TBHP + JQ1 group and TBHP + JQ1 + Compound c group, respectively. GAPDH was the loading control. *P < 0.05, **P < 0.01, ***P < 0.001. Data were presented as means ± SD.

As mentioned in previous studies, the AMPK-mTOR pathway is crucial in the modulation of autophagy (Dai et al., 2017; Ma et al., 2020). Therefore, to evaluate whether the induction of autophagy resulting from Brd4 inhibition is mediated by the AMPK-mTOR axis, mice and neurons were co-treated JQ1 and Compound c. After treatment with JQ1, increased levels of p-AMPK and p-ULK1, and decreased levels of p-mTOR were observed in injured mice by western blot analysis, while treatment with Compound c blocked the JQ1-mediated regulation of AMPK and mTOR (Figures 8A–D). Immunofluorescence staining of p-AMPK and NeuN also indicated that JQ1 treatment enhances the level of p-AMPK in neurons (Supplementary Figures 4A,B). Subsequently, Compound c was observed to abolish the JQ1-mediated enhancement of LC3 II/I activation and the degradation of p62, as well as the associated protection against apoptosis and oxidative stress (Figures 8E–I). Similarly, immunofluorescence staining results also showed that Compound c intervention attenuated the intensity of LC3 and increased the level of p62 (Figures 8J,K and Supplementary Figures 4C–E) in JQ1-treated SCI mice. In cultured neurons, Compound c prevented the fusion of LC3 and Lamp2 (Figures 8L,M). Taken together, these results demonstrated that the AMPK-mTOR pathway is involved in the JQ1-mediated activation of autophagy in neurons.

## Discussion

**FIGURE 9:**
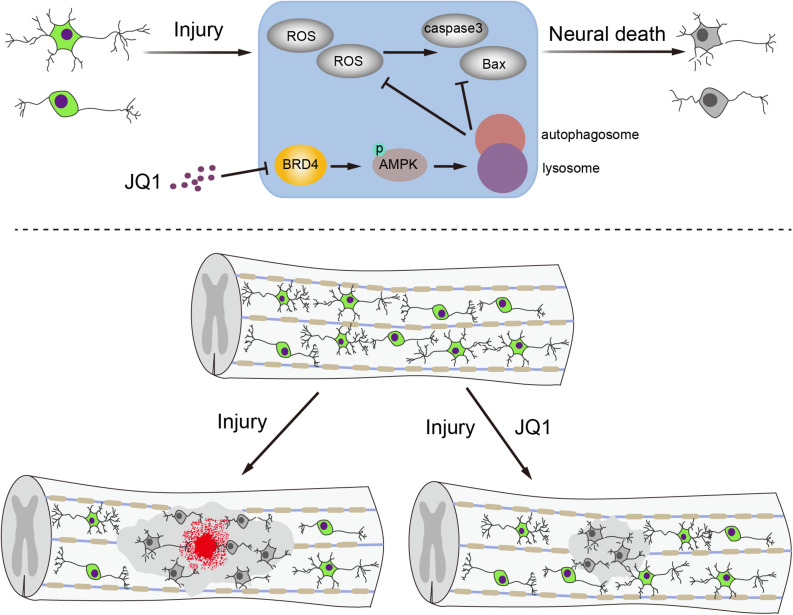
A potential mechanism of by which inhibition of Brd4 by JQ1 decreases oxidative stress and apoptosis. Inhibition of Brd4 by JQ1 promotes functional recovery through activating autophagy and restoring autophagic flux in a AMPK-mTOR-ULK1 dependent manner.

Apart from primary injury, neurons are particularly susceptible to secondary molecular events after SCI, including oxidative stress and cell apoptosis. Due to the poor regenerative capacity of neurons, inhibition of the secondary response after SCI to maintain neuron survival is a potential strategy for recovery after SCI (Wang et al., 2018; Li et al., 2019; Ohtake et al., 2019). In the present study, we observed that the expression of Brd4 increased around lesion sites after SCI, primarily in neurons. Pharmacological inhibition of Brd4 by JQ1 promotes functional recovery by activating autophagy and restoring autophagic flux, which subsequently attenuates oxidative stress and inhibits apoptosis (Figure 9). In the present study, we provide insights into the effects and potential molecular mechanisms of Brd4 inhibition by JQ1 and demonstrate the value of JQ1 for the clinical treatment of SCI.

Considering its potentially potent effects on transcriptional and epigenetic regulation, Brd4 has been investigated in various disease models. Brd4 was first shown to control the cell cycle and cell identity determination, with this protein being regarded as an essential regulator for maintaining cancer progression and metastasis and a promising target for anticancer treatment (Yang et al., 2008; Liu et al., 2014; Donati et al., 2018). In addition, the results from an increasing number of studies has shown that Brd4 is involved in the development of pulmonary fibrosis (Tian et al., 2016), cardiac hypertrophy (Spiltoir et al., 2013), osteoarthritis and intervertebral disk degeneration (An et al., 2018; Wang et al., 2019b). In the central nervous system, Brd4 is believed to be highly associated with cerebellar neurogenesis, as inhibition of Brd4 blocked learning and resulted in memory deficits (Korb et al., 2015; Penas et al., 2019). In contrast, a previous study demonstrated that inhibition of Brd4 is able to enhance brain plasticity and improve cognitive performance in an animal model of Alzheimer’s disease (Magistri et al., 2016). A recent study also suggested that inhibition of Brd4 is beneficial for the improvement of neurological deficits induced by stroke (Demars et al., 2019). Furthermore, Brd4 has been revealed to be an important factor in the regulation of the inflammatory response, as Brd4 inhibition by specific molecule could repress cytokine production and inflammation to promote functional recovery after SCI (Rudman et al., 2018; Sánchez-Ventura et al., 2019; Wang et al., 2019a). Consistent with previous reports, our results also showed that increased Brd4 expression is associated with the progression of SCI.

Of note, several small-molecule inhibitors of Brd4 have been identified and widely used in published studies, with JQ1, a cell permeable and efficient Brd4 inhibitor, used in the current study. JQ1 has been previously shown to improve arthritis, heart remodeling, Alzheimer’s disease and stroke by inhibiting Brd4 (Spiltoir et al., 2013; Magistri et al., 2016; An et al., 2018; Demars et al., 2019). In line with the results of these studies, we showed that inhibition of Brd4 by JQ1 has a pronounced positive effect on neural recovery after SCI by activating autophagy, conferring resistance to oxidative stress and apoptosis. This results suggested that JQ1 is a potentially important therapeutic agent. However, JQ1 has been observed to have potential toxicity (Walsh et al., 2019). The results of the present study showed that a high dose of JQ1 affects the growth of neurons to a certain extent. Therefore, we used a relatively safe concentration of JQ1 that was effective for maintaining neuronal homeostasis in response to injury stimulation.

Following trauma, the release of large amounts of reactive oxygen species (ROS) will disrupt the redox balance in cells and cause oxidative death in spinal cord neurons. Interestingly, Brd4 expression has been reported to be markedly increased in cardiomyocytes subjected to oxidative stress, indicating that oxidative stress induced by Brd4 is involved in cardiac hypertrophy (Zhu et al., 2020). In addition, recent studies have proven that inhibition of Brd4 by JQ1 or Brd4 knockdown significantly enhances the plasma levels of antioxidant enzymes and reduces lipid peroxidation, suggesting that JQ1 exerts a protective effect in augmenting antioxidant levels and protecting against oxidative stress (Michaeloudes et al., 2014; Chatterjee and Bohmann, 2018). This beneficial effect of Brd4 inhibition was also proven in our present study. Similar to previous studies, we observed that Brd4 expression is elevated in neurons using a ROS donor, and JQ1 treatment promotes the increased expression of HO-1 and SOD1, both of which have been identified as effective antioxidant enzymes. In addition, oxidative stress is important in promoting apoptosis, and the occurrence of neural apoptosis results in irreversible neural tissue loss both in primary and adjacent injury sites after SCI (Andrabi et al., 2020). In the present study, the results suggested that the inhibition of Brd4 by JQ1 maintains the neural homeostasis and survival of injured spinal cord tissue.

It is well-accepted that neurons are especially sensitive to autophagy dysfunction or defects. Previous studies have revealed that activation of autophagy is a self-protective process in response to various traumatic pathological factors by degrading and recycling excess or severely damaged macromolecules and organelles in central neural system diseases (Sarkar et al., 2014; Galluzzi et al., 2016). However, owing to the extreme changes in the intracellular microenvironment after SCI, lysosomes are impaired and cannot fuse with autophagosomes in neuron, with the accumulation of dysfunctional autophagosomes exacerbating neural death (Sarkar et al., 2014; Zheng et al., 2019). The relationship between Brd4 and autophagy has been observed, with a previous study showing thatBrd4 is a repressor of autophagy and lysosomal-related protein expression under normal conditions (Sakamaki et al., 2017). Consistent with this finding, Brd4 knockdown can increase autophagosome formation, lysosomal protein levels and the activity of lysosomal enzymes, and JQ1 treatment also increased the levels of autophagy, whereas this does not occur in the absence of Brd4, suggesting that JQ1-induced autophagy is required for Brd4 inhibition (Sakamaki and Ryan, 2017). In our present study, we observed that JQ1 treatment results in increased autophagy activity and promotes the fusion of autophagosomes and lysosomes to promote autophagic flux restoration. In addition, after inhibiting autophagy by 3-MA treatment, the effect of JQ1 in preventing oxidative stress and apoptotic activity and improving functional recovery after SCI was reversed. Therefore, autophagy is an important mechanism associated with JQ1-mediated protection in neurons.

With a clear role of JQ1 established in positively regulating autophagy, we focused on identifying the upstream mechanism associated with JQ1-mediated autophagy. More recently, a study has shown that the repression of autophagy by Brd4 could be ameliorated by AMPK-Sirt1 signaling when cells are under nutrient deprivation (Sakamaki et al., 2017). In addition, Brd4 inhibition by JQ1 can maintain the phosphorylation of RPS6KB/p70S6K, a substrate of the amino acid sensor mTORC1, which is a crucial factor involved in autophagy modulation (Wen and Klionsky, 2017). In acute myeloid leukemia (AML) stem cells, JQ1 has been reported to be associated with the modulation of cytoprotective autophagy by activating the AMPK-ULK1 axis (Jang et al., 2017). The results of the present study showed thatJQ1 treatment significantly increased the phosphorylation of AMPK and ULK1, decreasing the phosphorylation of mTOR. Based on the results using an AMPK inhibitor, the mechanism of increased autophagy in neurons treated with JQ1 is the consequence of the regulation of the AMPK axis.

There are several limitations of the current study that should be addressed in future investigations. For instance, mice were treated with a single dose of JQ1 directly after injury, while the optimal dose and duration time of treatment still requires further study. In addition, some experiments of the current study were performed with PC12 cells, and although this neural cell line has been commonly used *in vitro* for investigation of neurological diseases, the use of primary neurons would be more informative. As previously mentioned, Brd4 inhibition is associated with the regulation of the inflammation response, and intraperitoneal injection of JQ1 may also exert this effect on glial cells and infiltrative inflammation-related cells to improve functional recovery after SCI (Wang et al., 2019b; Brasier and Zhou, 2020). Furthermore, in consideration of the complex of autophagy regulation, attributing this regulation to AMPK-mTOR may be one-sided. Finally, we note that JQ1 may be involved in inhibiting other forms of BET proteins, and inhibition of Brd4 using a viral vector or by gene knockdown instead of JQ1 treatment would be more persuasive.

In summary, the results of the present study revealed that inhibition of Brd4 by JQ1 activates the AMPK-mTOR-ULK1 signaling pathway, resulting in augmentation of autophagy and restoration of autophagic flux in neurons, thereby attenuating oxidative stress, reducing neural apoptosis and improving functional recovery after SCI. Therefore, the inhibition of Brd4 by JQ1 represents a novel potential therapeutic approach for improving functional recovery after SCI.

## Data Availability Statement

The raw data supporting the conclusions of this article will be made available by the authors, without undue reservation, to any qualified researcher.

## Ethics Statement

The animal study was reviewed and approved by Animal Care and Use Committee of Wenzhou Medical University (wydw2018–0043).

## Author Contributions

YL designed the research, analyzed the data, and wrote the manuscript. JX performed the research. JZ and JL contributed new reagents or analytic tools. YW modified the manuscript. XW approved the final version and submitted. All authors contributed to the article and approved the submitted version.

## Conflict of Interest

The authors declare that the research was conducted in the absence of any commercial or financial relationships that could be construed as a potential conflict of interest.

## Supplementary Material

FIGURE S1 JQ1 attenuates oxidative stress in TBHP treated PC12 cells. **(A–C)** Western blotting and quantification of SOD1 and HO-1 expression in each group, cells of above groups were respectively treated with JQ1 (200 nM), TBHP (50 μM), JQ1 + TBHP for 6 h, *n* = 5. GAPDH was the loading control. ^∗^*P* < 0.05, ^∗∗^*P* < 0.01, ^∗∗∗^*P* < 0.001. Data were presented as means ± SD.

FIGURE S2 JQ1 enhances the level of Atg12-Atg5 conjugated level in neurons. **(A,B)** Western blotting and quantification of Atg12-Atg5 expression in primary cortical neurons treated with 200 nM JQ1 for 6 h, *n* = 5. GAPDH was the loading control. ^∗^*P* < 0.05, ^∗∗^*P* < 0.01, ^∗∗∗^*P* < 0.001. Data were presented as means ± SD.

FIGURE S3 JQ1 activates autophagy in PC12 cells. **(A–C)** PC12 cells were respectively treated with JQ1 (200 nM), TBHP (50 μM), JQ1 + TBHP for 6 h, LC3 and p62 expression were detected by western blotting and statistically quantized, *n* = 5. GAPDH was the loading control. ^∗^*P* < 0.05, ^∗∗^*P* < 0.01, ^∗∗∗^*P* < 0.001. Data were presented as means ± SD.

FIGURE S4 Compound c treatment inhibits JQ1 induced autophagy activation. **(A,B)** Immunofluorescence staining for the co-localization of p-AMPK (green) and NeuN (red) and quantitative analysis in the each group of mice at 3 days after SCI. **(C–E)** Immunofluorescence stained with LC3 and p62 and quantification of intensity in the each group of spinal cord at 3 days after SCI. *n* = 5. Scale bar = 50 μm, scale bar (enlarged) = 10 μm. ^∗^*P* < 0.05, ^∗∗^*P* < 0.01, ^∗∗∗^*P* < 0.001. Data were presented as means ± SD.
