# Supplementary figures and images for: Inhibition of Brd4 by JQ1 Promotes Functional Recovery From Spinal Cord Injury by Activating Autophagy
Source: Front Cell Neurosci. 2020 Sep 2;14:555591. doi: 10.3389/fncel.2020.555591 (PMC7493001; doi:10.3389/fncel.2020.555591)

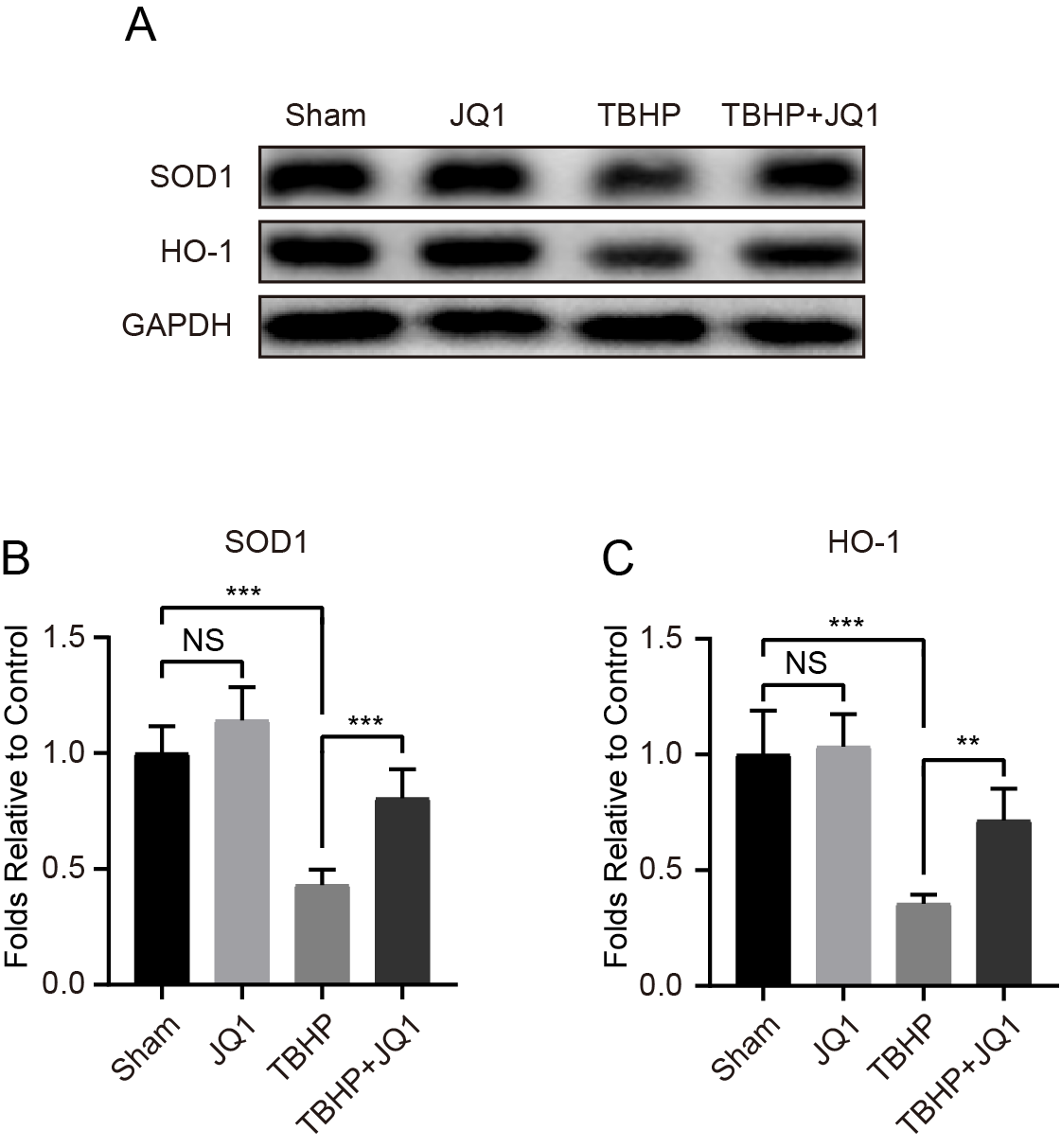

Supplement: FIGURE S1 — JQ1 attenuates oxidative stress in TBHP treated PC12 cells. (A–C) Western blotting and quantification of SOD1 and HO-1 expression in each group, cells of above groups were respectively treated with JQ1 (200 nM), TBHP (50 μM), JQ1 + TBHP for 6 h, n = 5. GAPDH was the loading control. ∗P < 0.05, ∗∗P < 0.01, ∗∗∗P < 0.001. Data were presented as means ± SD. [file Image_1.TIF]

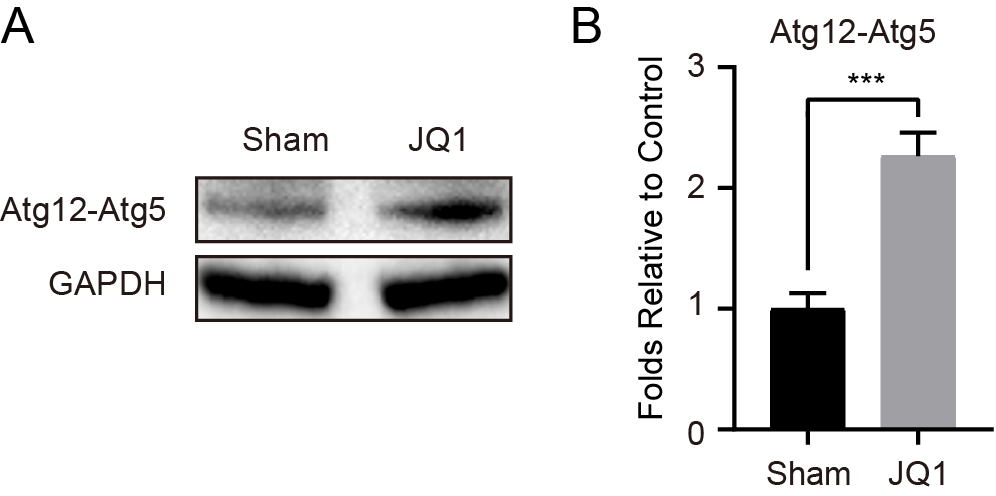

Supplement: FIGURE S2 — JQ1 enhances the level of Atg12-Atg5 conjugated level in neurons. (A,B) Western blotting and quantification of Atg12-Atg5 expression in primary cortical neurons treated with 200 nM JQ1 for 6 h, n = 5. GAPDH was the loading control. ∗P < 0.05, ∗∗P < 0.01, ∗∗∗P < 0.001. Data were presented as means ± SD. [file Image_2.TIF]

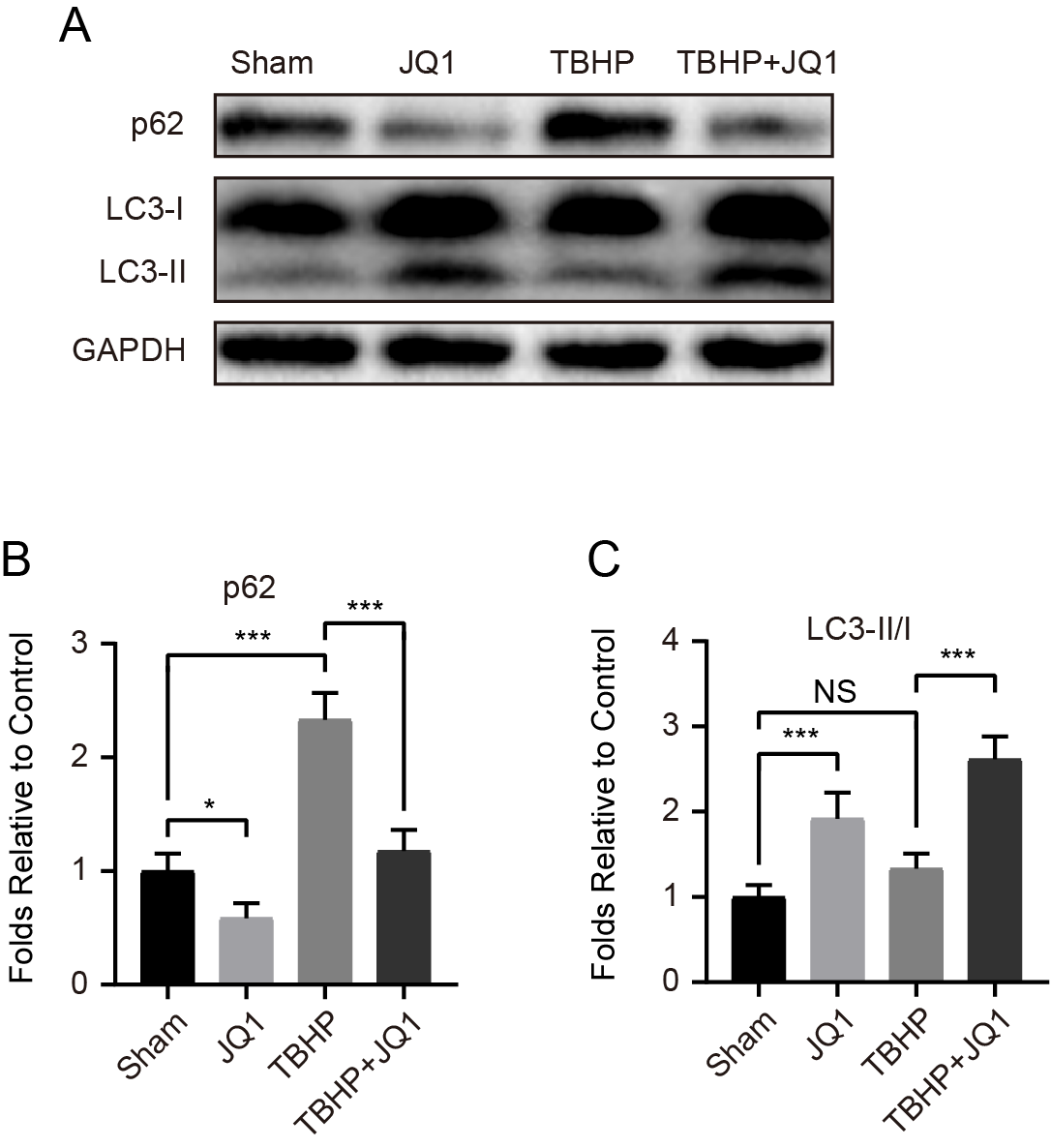

Supplement: FIGURE S3 — JQ1 activates autophagy in PC12 cells. (A–C) PC12 cells were respectively treated with JQ1 (200 nM), TBHP (50 μM), JQ1 + TBHP for 6 h, LC3 and p62 expression were detected by western blotting and statistically quantized, n = 5. GAPDH was the loading control. ∗P < 0.05, ∗∗P < 0.01, ∗∗∗P < 0.001. Data were presented as means ± SD. [file Image_3.TIF]

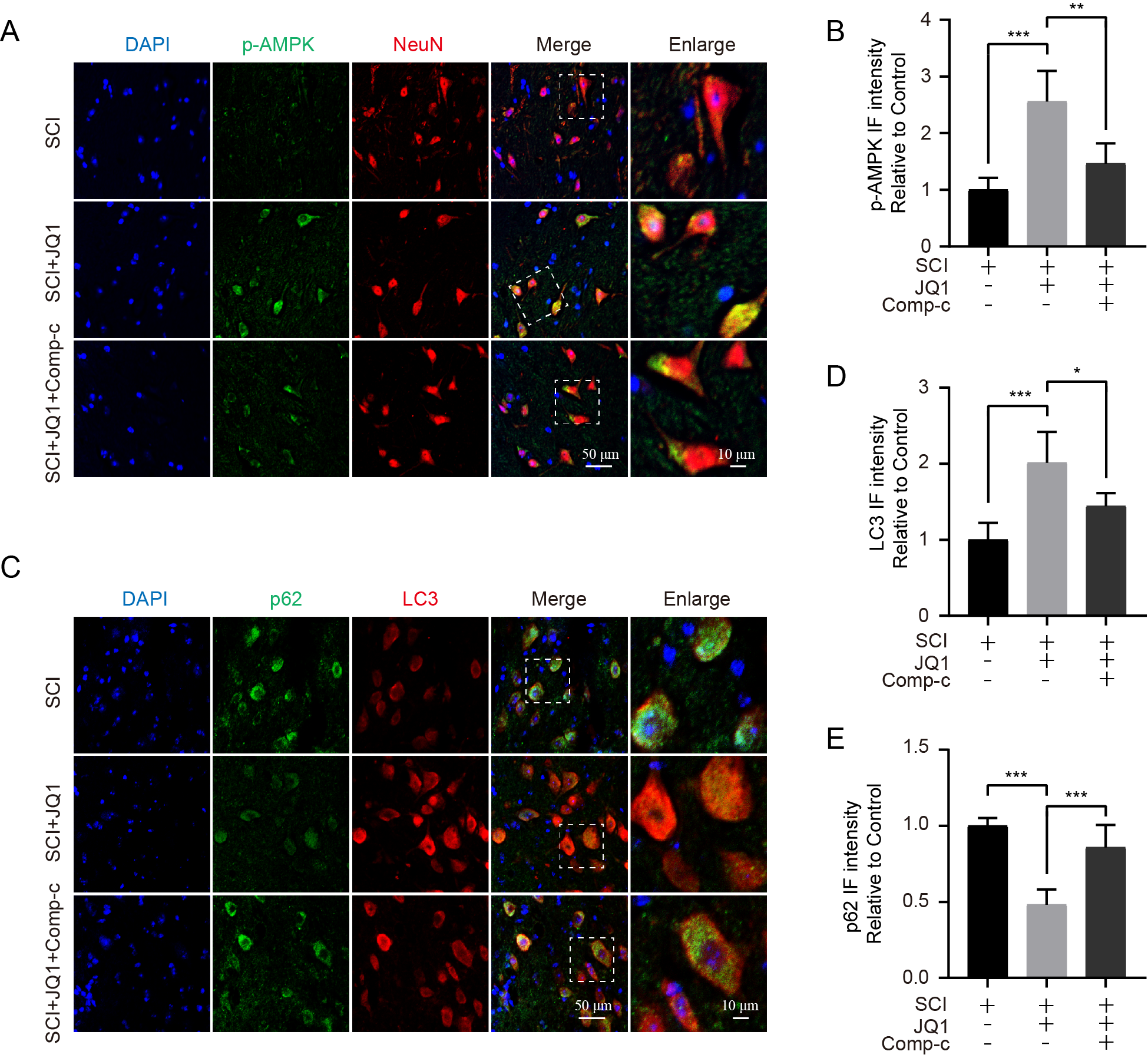

Supplement: FIGURE S4 — Compound c treatment inhibits JQ1 induced autophagy activation. (A,B) Immunofluorescence staining for the co-localization of p-AMPK (green) and NeuN (red) and quantitative analysis in the each group of mice at 3 days after SCI. (C–E) Immunofluorescence stained with LC3 and p62 and quantification of intensity in the each group of spinal cord at 3 days after SCI. n = 5. Scale bar = 50 μm, scale bar (enlarged) = 10 μm. ∗P < 0.05, ∗∗P < 0.01, ∗∗∗P < 0.001. Data were presented as means ± SD. [file Image_4.TIF]
